# A Tat/Rev Induced Limiting Dilution Assay to Measure Viral Reservoirs in Non-Human Primate Models of HIV Infection

**DOI:** 10.1038/s41598-019-48354-3

**Published:** 2019-08-19

**Authors:** Ines Frank, Arpan Acharya, Nanda K. Routhu, Meropi Aravantinou, Justin L. Harper, Stephanie Maldonado, Maria Sole Cigoli, Stanka Semova, Svetlana Mazel, Mirko Paiardini, Nina Derby, Siddappa N. Byrareddy, Elena Martinelli

**Affiliations:** 1 Center for Biomedical Research, Population Council, New York, NY USA; 2 Department of Pharmacology and Experimental Neurosciences, University of Nebraska Medical Center, Omaha, USA; 3 Yerkes National Primate Research Center, Emory University, Atlanta, Georgia USA; 4 Flow Cytometry Resource Center, Rockefeller University, New York, NY USA

**Keywords:** Experimental models of disease, Infection, Preclinical research

## Abstract

The establishment of latent infection and poorly characterized viral reservoirs in tissues represent major obstacles to a definitive cure for HIV. Non-human primate (NHP) models of HIV infection are critical to elucidate pathogenic processes and an essential tool to test novel therapeutic strategies. Thus, the availability of novel assays to measure residual viral replication and reservoirs in NHP models may increase their utility in the search for an HIV cure. We developed a tat/rev induced limiting dilution assay to measure the frequency of CD4^+^ T cells that express multiply-spliced(ms)_SIV RNA in presence and absence of stimulation. We validated the assay using cell lines and cells from blood and lymph nodes of SIV infected macaques. *In vitro*, SIV/SHIV TILDA detects only cells expressing viral proteins. In SIV/SHIV-infected macaques, CD4^+^ T cells that express msSIV/SHIV RNA (TILDA data) were detected also in the setting of very low/undetectable viremia. TILDA data were significantly higher after stimulation and correlated with plasma viral load (pVL). Interestingly, TILDA data from early cART initiation correlated with peak and AUC pVL post-cART interruption. In summary, we developed an assay that may be useful in characterizing viral reservoirs and determining the effect of HIV interventions in NHP models.

## Introduction

Despite the major success of combined antiretroviral therapy (cART) in suppressing HIV replication and preventing disease progression, HIV infection persists due to the early establishment of the viral reservoir, which is not eliminated by any of the available therapies and is considered the major obstacle to HIV cure^1–3^. Following cessation of cART, plasma viremia rebounds within weeks in most of the HIV infected individuals^2^. The viral reservoir in the peripheral blood of HIV infected, cART treated individuals has been extensively characterized and persists for decades within resting memory CD4^+^ T cells^4–6^. Mechanisms of persistence include long term survival of these resting cells as well as physiologic processes of immunologic memory that drive clonal expansion of infected cells^4,5,7^.

Notably, despite the advances in understanding the viral reservoir in blood, viral reservoirs in tissues remain poorly characterized. Ethical and practical considerations limit studying these tissue reservoirs in humans and also prevent the evaluation of novel therapeutic strategies that may present unnecessary risks to otherwise healthy individuals. Non-human primate (NHP) models have been critical to a better understanding of the establishment of the viral reservoir and the potential effect of novel therapeutic strategies^8^. A permanent replication competent viral reservoir was shown to form as early as 3 days post-infection in an SIVmac251 model^9^. Moreover, studies in SIV infected macaques helped establish a role for CD8^+^ T cells in maintenance of viral control during cART^10^ and the potential beneficial effects of IL-21 and probiotic therapies on the reduction of the viral reservoir^11,12^. Importantly, studies in NHP helped investigating viral reservoirs in tissues, such as lymph nodes germinal centers^13–15^, gut-associated lymphoid tissues^16^ and the brain^17,18^.

A variety of techniques are currently employed to characterize the viral reservoir in HIV infected humans^19^. They include assays that directly measure cell-associated (CA)-viral DNA or RNA with more recent assays including the ability to distinguish ‘intact proviruses’ with the theoretical capability to replicate, from defective proviruses^20^. This is a notable advancement compared to techniques that measure total viral CA-DNA that greatly overestimate a viral reservoir constituted mostly by defective proviruses with deletions and hypermutations^20^. Other methods include several variants of culture-based assays known as quantitative viral outgrowth assays (QVOA)^21^. These assays have the advantage of quantifying only replication competent virus^22,23^. However, they are labor intensive and underestimate the size of the HIV reservoir because of suboptimal induction and/or inadequate propagation of all replication-competent viruses *in vitro*^24^. Finally, techniques that measure induced levels of viral RNA or proteins, while not a direct reflection of replication competent viral reservoir, may better reflect immunologically relevant/antigen producing reservoir compartments^25^. Among these techniques there is the tat/rev induced limiting dilution assay (TILDA), which measures the frequency of CD4^+^ T cells producing viral multiply-spliced RNA (msRNA) transcripts either in absence of stimulation or following stimulation with a potent mitogens (phorbol 12-myristate 13-acetate -PMA - and ionomycin) using serial dilutions of input CD4^+^ T cells^26^. This strategy gives estimates of viral reservoir that are between the underestimates of the QVOA and the overestimates of the cell associated viral DNA and unspliced (us)RNA^19^. Indeed, because tat/rev transcripts are generated after splicing of full-length viral transcripts, TILDA reduces the likelihood of measuring proviruses with large internal deletions^27^. Moreover, major reversible inhibition steps to HIV expression in latently infected cells were shown to reside in blocks to elongation, maturation and multiple splicing, and not in the initiation of transcription^28^. On the other hand, tat/rev transcripts are required (but not sufficient) for the production of functional viral particles and the HIV TILDA does not bypass completely the possibility of measuring defective proviruses^29^.

Thus, the HIV TILDA seems to, at the same time, underestimate and overestimate the size of the HIV reservoir. This is, respectively, because of the higher likelihood of a strong stimuli such as PMA/ionomycin to more readily kill infected cells with intact proviruses than uninfected cells or cells with defective proviruses^20^ and because a single round of stimulation does not reactivate the entire pool of replication competent proviruses^24^. Nonetheless, the HIV TILDA has the important advantages of being relatively fast, high throughput and requires a relatively small number of cells compared to culture-based assays^19^.

A similar variety of assays to measure the viral reservoir are used in NHP studies. However, their use in NHP is not always thoroughly validated and the inevitable differences between cART treated HIV infection and NHP models are rarely taken into account. Certain culture-based assays in particular, such as the QVOA, require a very high number of cells in order to make cross-samples comparisons with adequate statistical power, which is unfeasible and unreasonably expensive in NHP studies. Thus, we adapted the HIV TILDA assay to NHP models of HIV infection. We particularly focused on developing and validating an SIV/SHIV TILDA assay that could be used with the SIVmac251/mac239 and SHIV_AD8OE_ viruses, since they are widely employed in NHP studies focused on evaluating novel therapeutic strategies for HIV. We validated the assay and determined that it may be useful in predicting the outcome of interventions during cART. In summary, the SIV/SHIV TILDA is an informative additional tool in the investigation of viral reservoirs in NHP models to complement other novel, sensitive strategies such as the p27 Simoa^25^ and *in situ* hybridization approaches^30^.

## Results

### Establishment of the SIV-TILDA assay

**Figure 1:**
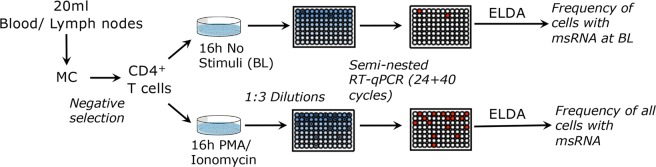
Schematic of SIV/SHIV TILDA assay. CD4^+^ T cells are isolated by negative isolation with beads from blood or lymph nodes mononuclear cells (MC) and incubated for 16 h with PMA/ionomycin or in absence of stimuli. Then cells are counted and distributed in 1:3 dilutions in 22 replicate wells per dilution. A semi-nested RT-qPCR is run directly on the cells and the frequency of cells producing msRNA is calculated using the extreme limiting dilution assay (ELDA) software.

We sought to develop an assay that detects tat/rev msRNA in maximally activated CD4^+^ T cells from SIV and SHIV infected rhesus macaques. We adapted the well-established and validated HIV TILDA assay^26^ to the SIV and SHIV models, determined the sensitivity and specificity of the SIV-TILDA and its applicability. As in the HIV TILDA, isolated CD4^+^ T cells are stimulated with PMA and ionomycin, which have been shown to induce high levels of viral production in several primary cell models of HIV latency^31^. After stimulation, cells are distributed in 22 replicate wells and a nested-PCR amplifying tat/rev transcripts is directly performed without RNA extraction as in the HIV-TILDA. The frequency of cells producing msRNA is calculated using the maximum likelihood method via the ELDA (extreme limiting dilution assay) software^32^. As in the HIV-TILDA, the frequency of CD4^+^ T cells that spontaneously produce msRNA is measured at the same time by omitting the stimulation step as described in Fig. 1.

We validated the SIV-TILDA assay using the SIVmac239ΔNef model (SIVΔNef) and the corresponding SIVmac239 wild-type (wt) and SIVmac251 models by designing novel primers and probes that match the HIV regions used to detect the tat/rev msRNA in Pasternak *et al*.^33^. SIVmac251/mac239 viruses are common in models of SIV pathogenesis and for testing of novel therapeutic strategies. The SIVΔNef model was useful because the low replicative fitness of this virus *in vivo* during chronic infection allowed developing and testing the assay in low viremic, SIVΔNef infected animals. SHIV models are also starting to gain prominence in studies of pathogenesis and cure, especially for testing of therapeutic strategies involving bNAbs and immunogens based on HIV Env. Since HIV TILDA primers and probes can be used for monkeys infected with SHIV-SF126P3 (Martinelli unpublished), we validated the assay with the SHIV_AD8OE_ model. Infection with SHIV_AD8OE_ has been recently used for bNAbs-cure studies^34,35^. The assay requires a mix of the HIV TILDA and SIV-TILDA primers and probes and it was named SHIV-TILDA.

### SIV TILDA is Specific, Sensitive and Reproducible

**Figure 2:**
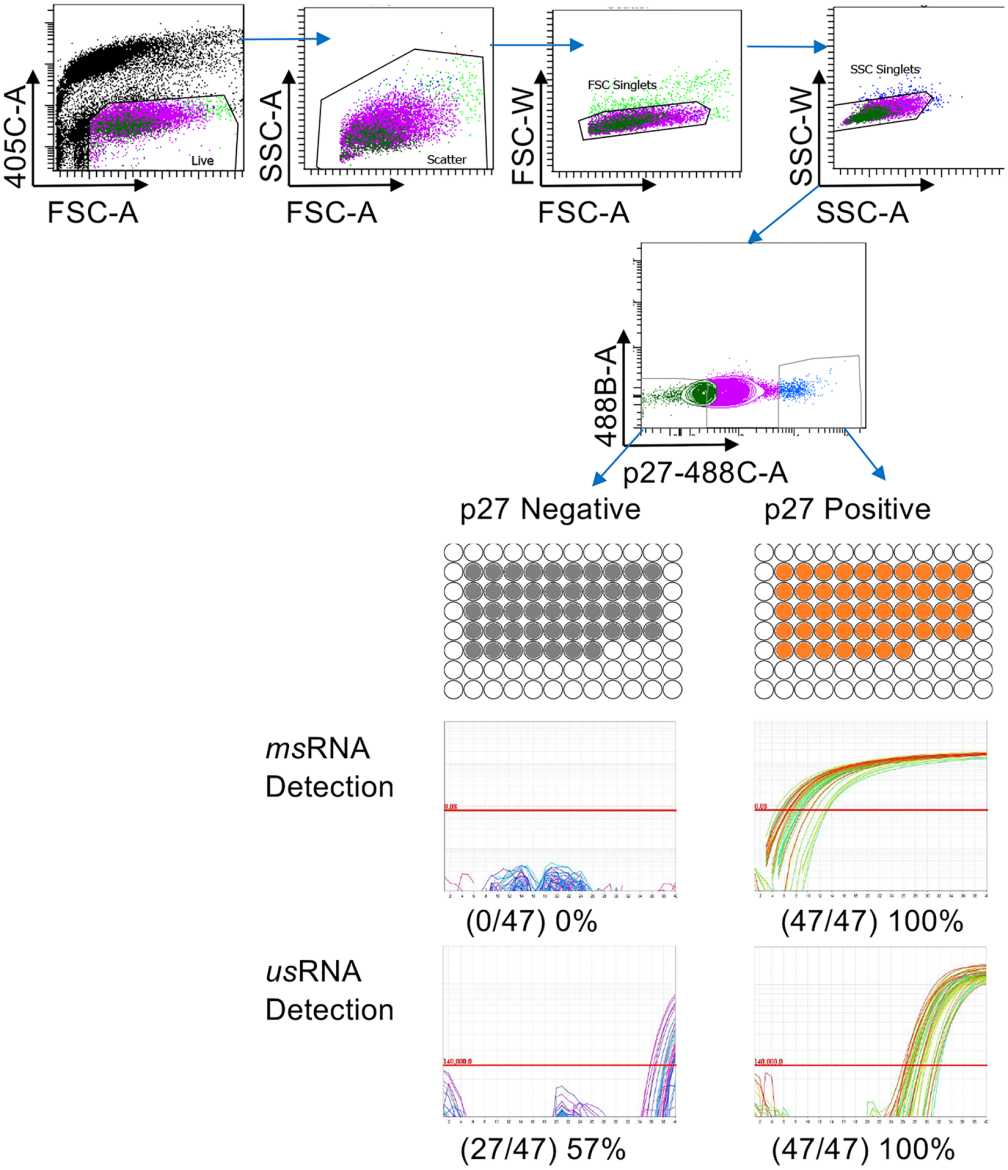
The SIV TILDA detects msRNA only in cells expressing also viral proteins. CEM cells were infected with SIVmac239 for 3 days and then p27+ and p27− cells were sorted on a BD FACS Aria II cell sorter. p27+ and p27− cells were distributed in 47 wells/each and TILDA semi-nested RTqPCR or *gag*RT-qPCR were run. Sorting gating strategy is shown above, while actual amplification curves from the assays are shown below next to the final results.

The HIV TILDA was developed as assay to quantify the viral reservoir based on the assumption that presence of tat/rev msRNA reflects the ability of a cell to produce virus^33^. In contrast, low amounts of HIV CA usRNA are detected in latently infected CD4^+^ T cells that do not produce replication competent virus^36,37^ and in PBMC from virally suppressed individuals on cART^38–40^. Thus, to validate the specificity of the SIV TILDA and verify its ability to detect only cells that produce replication competent virions, we infected CEMx174 cells with SIVmac239 *in vitro* for 3 days. We sorted cells expressing p27 intracellularly (p27+) from p27− cells and used our assay to detect tat/rev msRNA in wells containing 10 or 1 p27+ or p27− cells. In the SIV TILDA experiment with 10 cells/well, 47 out of 47 wells containing p27+ cells were positive both in the SIV TILDA assay and in the *gag*RT-qPCR assay for SIV *us*RNA. 47 out of the 47 p27− wells were negative in the SIV TILDA assay (Fig. 2). In contrast, 26 out of 47 p27− wells were positive in the *gag*RT-qPCR assay, indicating that the majority of the p27− cells were infected, but did not yet produce msRNA nor p27 protein (Fig. 2). In the 1 cell/well experiment, we confirmed these results although the fraction of positive cells was lower (0/47 p27− wells and 29/47 p27+ wells for the SIV TILDA, 4/47 p27− wells and 31/47 p27+ wells for the *gag*RT-qPCR) probably because not all wells contained a cell. These data confirm that the presence of msRNA is a specific surrogate for the production of SIV viral proteins in this *in vitro* system. Since acutely infected cells may not harbor many defective genomes, which tend to accumulate over time *in vivo*, we determined the specificity of the SIV TILDA in CD4^+^ T cells isolated from 2 SIVmac251 chronically infected macaques. After 3 days from PMA/Ionomycin activation in presence of T20, the CD4^+^ T cells were sorted directly in PCR plates in wells containing p27+ (1 cell/well) or p27− cells (10 cells/well) and the SIV TILDA was run on several replicates/condition. Even in this *ex vivo* system, the SIV TILDA was able to detect only p27+ wells (23/25), while all p27− wells resulted negative (0/46) to the nested RT-qPCR. This result suggests that the detection of msRNA from defective genomes with this assay is negligible.

**Figure 3:**
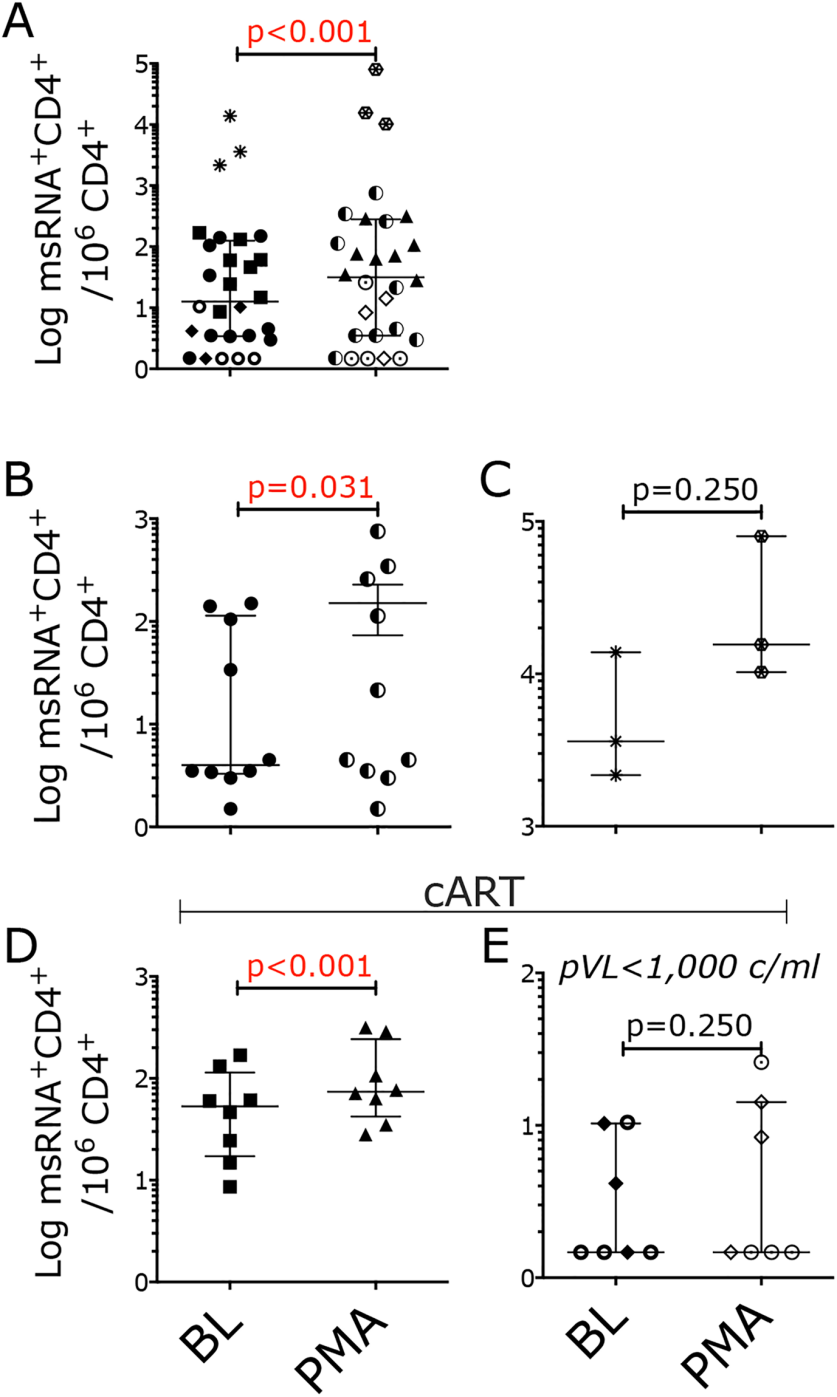
SIV TILDA measures the frequency of CD4^+^ T cells producing msRNA in SIV infected macaques. (**A**–**E**) Frequency of CD4^+^ T cells expressing msRNA per 10^6^ CD4^+^ T cells in the unstimulated (BL) and stimulated (PMA) conditions (TILDA data). (**A**) TILDA data from all SIV infected macaques. (**B**) TILDA data from SIV∆Nef infected macaques (circles) (**C**) TILDA data from SIVmac251 infected macaques (asterisks and hexagons) (**D**) TILDA data from SIVmac239Nef_stop infected cART treated macaques (squares and triangles) (**E**) TILDA data from SIVmac239 infected, cART treated macaques with pVL < 1000 or undetectable (diamonds and open circles). All data are from blood with the exception of the open circles in E, which are from LNs. Data in the BL and PMA conditions were compared with the Wilcoxon matched pairs signed-rank test (p < 0.05 was considered significant). Bars represent median ± IQR.

In order to determine the sensitivity of the semi-nested RT-qPCR of the SIV-TILDA, 10-fold dilutions from 1000 to 1 p27+, SIVmac251 infected macaque CD4^+^ T cells were mixed with 10,000 uninfected macaque CD4^+^ T cells in 10 replicates/ dilution. The semi-nested RT-qPCR of the SIV-TILDA was run on all the replicates in each dilution. The assay amplified msRNA in all replicates in all dilutions, but for the highest dilution with 1 p27+ cells within 10,000 p27− cells, where only 1 out of 10 replicates was positive. Thus, the sensitivity of the semi-nested RT-qPCR used for the SIV TILDA is 1 p27+ cell within 10,000 p27− cells. As for the HIV TILDA, the theoretical dynamic range of the SIV TILDA is >3Log10 and depends on the ELDA calculations with the minimal and maximal detectable values changing according to the choice of number of cells in the starting dilution. Typically, the HIV TILDA starts with 18,000 cells and it is performed with 3-fold dilutions down to 1000 cells/ well. With the 22 replicates/ dilution used in our assay the minimal and maximal values are, respectively, 1.49 and 3096 (0.17 Log–3.5 Log) cells/10^6^ CD4^+^ T cells. However, we found that this dynamic range is useful only for macaques with a plasma viral load (pVL) < 5 × 10^3^ copies/ml. In macaques with pVL ranging from 5 × 10^3^ to 5 × 10^4^ copies/ml starting with 9000 cells and performing 3-fold dilutions down to 300 cells will gives results more toward the center of the dynamic range - now between 3.47 and 10,256 (0.5 Log–4 Log) cells/10^6^ CD4^+^ T cells - than starting with 18,000 cells. In macaques with pVL ranging from 5 × 10^4^ to 5 × 10^5^ copies/ml starting with 3000 cells and performing 3-fold dilutions down to 100 cells will give results more toward the center of the dynamic range - now between 10.49 and 30,488 (1 Log–4.5 Log) cells/10^6^ CD4^+^ T cells - than starting with 18,000 or 9,000 cells (as shown in Fig. 3, viremic animals with pVL above 5 × 10^4^ copies/ml have TILDA values between 3 Log and 4.5 Log). Changing the dynamic range of the assay according to the pVL of the macaque is useful in the SIV TILDA because we foresee the use of this assay in macaque models evaluating different therapeutic interventions that may not include a fully suppressive cART regiment and/or may need to investigate early stages post-cART initiation, when viral suppression is not yet complete. In contrast to the HIV TILDA assay, which is used in fully suppressed HIV infected individuals who have been for years under cART, the SIV TILDA assay may be used in the setting of low-medium viremia with higher spontaneous baseline production of msRNA and much higher msRNA production after PMA stimulation than in the HIV TILDA.

Finally, using samples obtained from two SIVmac251 infected macaques, we determined the robustness of the SIV TILDA i.e. inter- and intra- assay coefficients of variation. The frequency of CD4^+^ T cells producing msRNA spontaneously or upon PMA/ionomycin stimulation were measured in duplicate plates or in independent experiments performed by 2 different operators. The intra- and inter- assay CVs (unstimulated and PMA condition were averaged) were 9.7% and 14.5%, respectively.

### The SIV TILDA measures the frequency of CD4^+^ T cells with inducible msRNA in SIV infected macaques

We used the SIV TILDA to measure the frequency of CD4^+^ T cells producing tat/rev msRNA in samples from 3 SIVmac251 infected and 9 SIVΔNef infected, untreated macaques and from 15 SIVmac239 infected (8 infected with the SIVmac239_Nef_Stop_^41^) cART treated macaques (see Table 1 for a complete list).

**Table 1:** List of macaques used in the study.

Macaque	Sex	Virus	Week p.i.	Plasma VL	Sample Type	cART Regiment	Days after cART start
IJ49	M	SIVmac251	16	8,100,000	PBMC	n/a	n/a
IB80	M	SIVmac251	16	11,000,000	PBMC	n/a	n/a
IN44	M	SIVmac251	9	910,000	PBMC	n/a	n/a
GL03	M	SIV∆Nef	16	65	PBMC	n/a	n/a
HT20	M	SIV∆Nef	9	15	PBMC	n/a	n/a
JN70	M	SIV∆Nef	14	7,000	PBMC	n/a	n/a
JN70-R	M	SIV∆Nef	18	30,000	PBMC	n/a	n/a
JN27	M	SIV∆Nef	16	29,000	PBMC	n/a	n/a
IC50	M	SIV∆Nef	15	5,400	PBMC	n/a	n/a
IH05	M	SIV∆Nef	20	65	PBMC	n/a	n/a
JI44	M	SIV∆Nef	14	350	PBMC	n/a	n/a
HE49	M	SIV∆Nef	9	2,300	PBMC	n/a	n/a
IM98	M	SIV∆Nef	15	1,000	PBMC	n/a	n/a
REt15R	F	SIVmac239_Nef_Stop_	6	6857	PBMC	PMPA/FTC/L-870812	4
RFw15R	F	SIVmac239_Nef_Stop_	6	127365	PBMC	PMPA/FTC/L-870812	4
RHd15R	F	SIVmac239_Nef_Stop_	6	11635	PBMC	PMPA/FTC/L-870812	4
RKc15R	F	SIVmac239_Nef_Stop_	6	40385	PBMC	PMPA/FTC/L-870812	4
RGe15R	F	SIVmac239_Nef_Stop_	6	489923	PBMC	PMPA/FTC/L-870812	4
Rld16R	F	SIVmac239_Nef_Stop_	6	132347	PBMC	PMPA/FTC/L-870812	4
RLm15R	F	SIVmac239_Nef_Stop_	6	13603	PBMC	PMPA/FTC/L-870812	4
RMb15R	F	SIVmac239_Nef_Stop_	6	13267	PBMC	PMPA/FTC/L-870812	4
RLz8	F	SIVmac239	32	< 1,000	PBMC	PMPA/FTC/RAL/DRV	63
Rwp8	F	SIVmac239	33	< 1,000	PBMC	PMPA/FTC/RAL/DRV	70
RMq8	F	SIVmac239	36	< 1,000	PBMC	PMPA/FTC/RAL/DRV	91
Rzp11	F	SIVmac239	35	< 50	LN	PMPA/FTC/RAL/DRV	84
Rlp10	F	SIVmac239	33	< 50	LN	PMPA/FTC/RAL/DRV	70
Rkt9	F	SIVmac239	33	< 50	LN	PMPA/FTC/RAL/DRV	70
Rfw8	F	SIVmac239	33	< 50	LN	PMPA/FTC/RAL/DRV	70

5 different groups of macaques were used to develop and validate the SIV TILDA assays. Groups differed by virus used for infection, absence or presence of cART treatment and pVL. Detailed characteristics of each animals are listed. Animals used for SHIV_AD8OE_ studies are listed in Table S1.

Spontaneous production of msRNA as well as production of msRNA upon stimulation were detected in all viremic animals (Fig. 3A–D), in 2 out of 3 cART-treated animals with pVL < 1000 copies/ml (Fig. 3E, diamonds) and in 1 out of 4 lymph nodes (LNs) of cART-treated animals with undetectable pVL (Fig. 3E, circles). As expected, in all samples, the frequency of CD4^+^ T cells spontaneously producing tat/rev msRNA was always lower than the frequency of CD4^+^ T cells producing tat/rev msRNA upon PMA/ionomycin stimulation (Fig. 3A). The overall median frequency of cells producing msRNA in the unstimulated samples was 12.6 ± 3.4–125.2 cells/CD4^+^ T cells (median ± IQR; including all macaque groups), while in the stimulated samples was 31.58 ± 3.6–280.5 cells/CD4^+^ T cells (median ± IQR; including all macaque groups), a median 2.5-fold increase after stimulation (including all samples with detectable msRNA). These frequencies are, respectively, higher and lower than the median frequencies in the unstimulated and stimulated samples in the HIV TILDA assay in cART treated individuals, but lower than those from HIV viremic individuals^26^. This was expected since most of the macaques we used were viremic, but their viremia was lower than in untreated HIV patients because of poor viral fitness (SIVΔNef) or because of cART.

**Figure 4:**
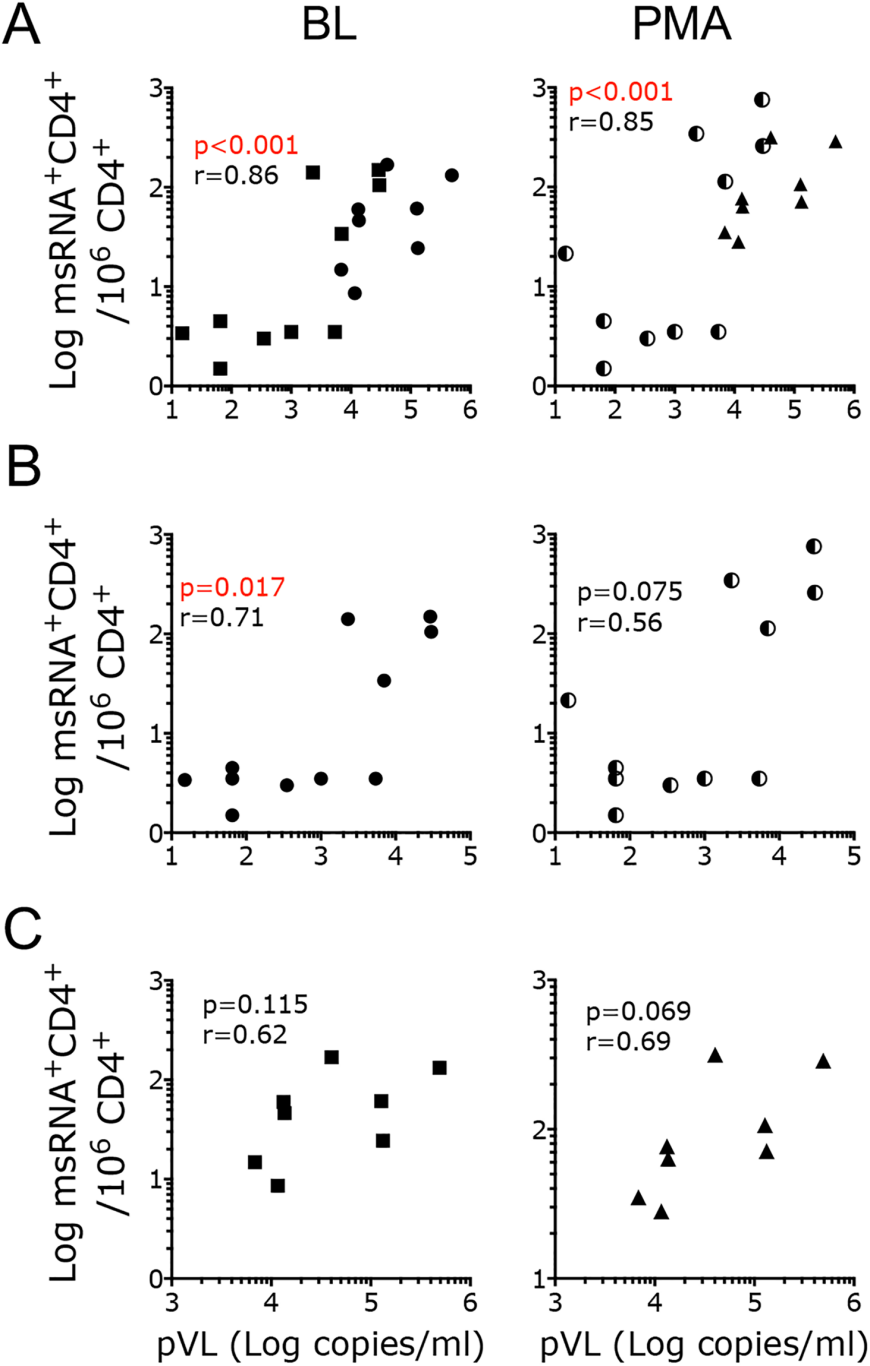
The frequency of CD4^+^ T cells producing msRNA in SIV infected macaques poorly correlates with pVL. (**A**–**C**) Frequency of CD4^+^ T cells expressing msRNA per 10^6^ CD4^+^ T cells in the unstimulated (BL) and stimulated (PMA) conditions is plotted against the plasma viral load at the time of the assay. (**A**) Shown are data from all SIV∆Nef and SIVmac239Nef_stop infected macaques. (**B**) Only TILDA data from SIV∆Nef macaques (circles) are correlated with pVL. (**C**) Only TILDA data from SIVmac239Nef_stop, cART treated macaques (squares for BL and triangles for PMA) are correlated with pVL. Correlation was calculated using the non-parametric Spearman test. No outlier was excluded. Spearman r and p values are shown (p < 0.05 was considered significant).

Notably, the frequency of CD4^+^ T cells producing tat/rev msRNA (spontaneously and upon stimulation) significantly correlated with pVL at the same time point only when data from all the samples were considered (Fig S1) or at minimum when samples from the SIVΔNef infected animals were grouped together with those from ART-treated viremic animals (Fig. 4A). However, when the different groups of animals were considered separately, the SIV TILDA data did not correlate or correlated very weakly with pVL (Fig. 4B,C).

### The frequency of CD4^+^ T cells producing tat/rev msRNA significantly correlates with viral rebound post-cART interruption

**Figure 5:**
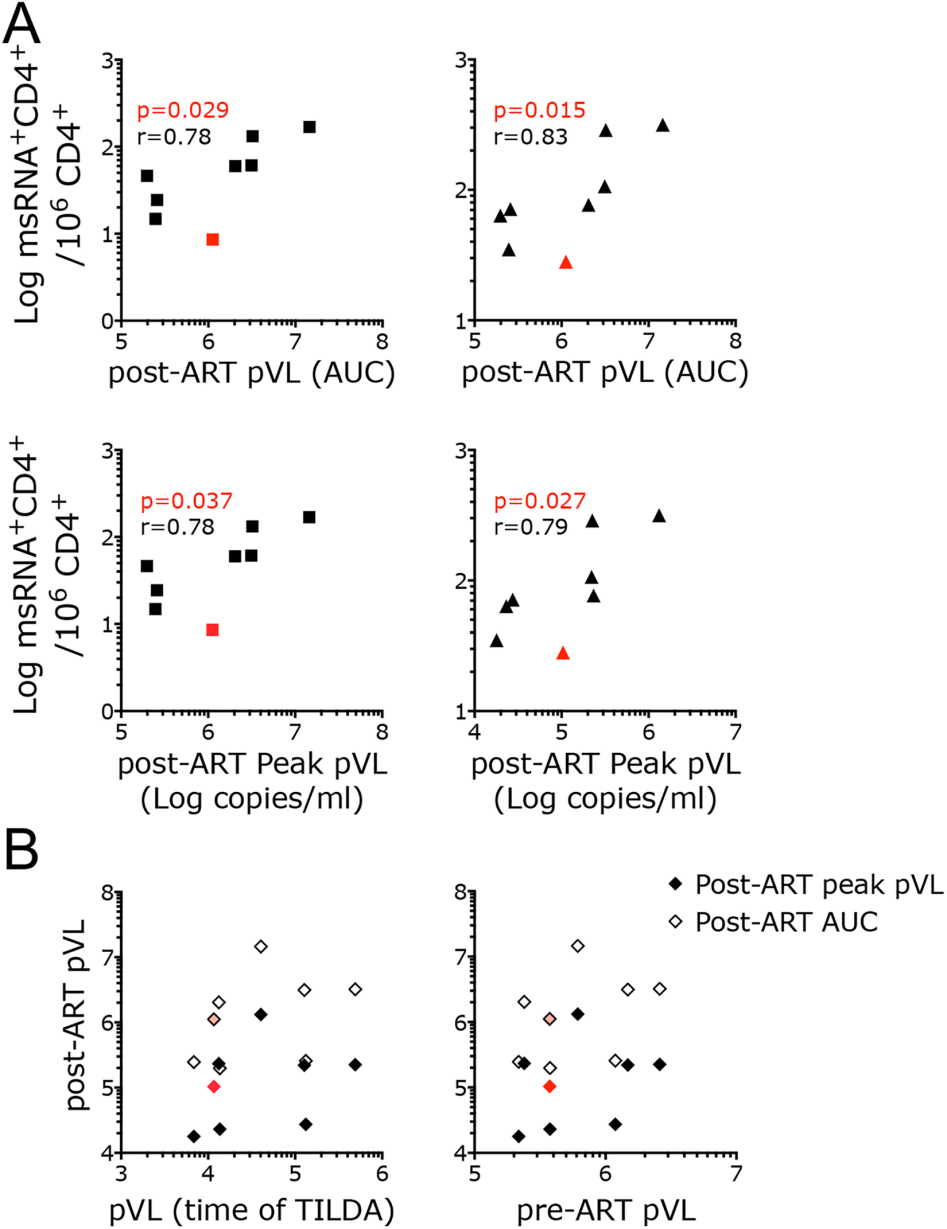
The frequency of CD4^+^ T cells producing msRNA soon after cART initiation correlate with post-ART pVL. (**A**) The frequency of CD4^+^ T cells producing msRNA at day 4 post-cART in the unstimulated condition (left; squares) and after PMA/ionomycin stimulation (right; triangles) are plotted against the AUC (upper row) or peak (lower row) pVL of the first 5 weeks post-cART interruption. (**B**) Peak (dark diamonds) and AUC pVL (open diamonds) for the first 5 weeks post-cART interruption are plotted against pVL at the time of the TILDA assay (4 days after cART initiation; left) or against pVL measured on the day of cART initiation. Correlations were calculated using the non-parametric Spearman test. All data were included. Spearman r and p values are shown (p < 0.05 was considered significant). The point in Red represents the sample with the lowest msRNA in both stimulated and unstimulated condition. This animal was the last one to rebound 3 weeks post-cART interruption.

Since in macaque models of HIV infection, especially in pilot studies, it is not always possible to treat macaques with cART for prolonged periods of time due to cost constrains and technical challenges, we asked if the frequency of CD4^+^ T cells producing tat/rev msRNA right after cART initiation could be indicative of response to treatment and predict the magnitude of viral rebound after cART interruption. This would suggest that the SIV-TILDA could be used to estimate the viral reservoir at least in SIV models similar to the one we investigated. As estimate of magnitude of viral rebound post-cART, we used the area under the curve (AUC) of the pVL within the first 5 weeks after cART interruption as well as peak pVL after interruption. Interestingly, the unstimulated and stimulated frequency of CD4^+^ T cells producing tat/rev msRNA were significantly associated with both measures of viral rebound post-cART (Fig. 5A). This is in contrast to the pVL at the time of cART initiation and pVL at the time of the SIV-TILDA assay (4 days after cART initiation) that did not correlate with viral rebound (post-ART pVL AUC and peak; Fig. 5B).

Time-to-viral rebound post-cART interruption is considered one of the best measurements of the size of the viral reservoir both in humans and macaques^4,19,42^. Unfortunately, in the 8 animals analyzed in the present study a definitive correlation between time-to-rebound and the frequency of CD4^+^ T cells expressing msRNA could not be assessed. This is because pVL was measured only weekly after cART interruption and all animals rebounded within the first 2 weeks, but for 1 animal. Interestingly, this last macaque that rebounded 3 weeks after cART interruption had the lowest frequency of CD4^+^ T cells expressing msRNA both in the unstimulated and stimulated condition (Fig. 5B in red). Of note, this animal had neither the lowest pre-cART pVL, nor the lowest pVL at the time of the TILDA assay (Fig. 5A).

### SHIV TILDA measures the frequency of CD4^+^ T cells with inducible msRNA in the lymph nodes of SHIV_AD8OE_ infected macaques

In order to investigate the use of HIV bNAbs for treatment or cure strategies, SHIV models with HIV envelope protein need to be employed. The SHIV_AD8OE_ model has been used in several recent studies investigating bNAbs therapeutic activity^34,35^, because of its multiple properties typical of pathogenic HIV-1 isolates^43^. Thus, after verifying the alignment of the HIV and SIV TILDA primers and probes, we adapted our SIV TILDA assay to the SHIV_AD8OE_ model by substituting the reverse primer and probe with those from the HIV TILDA^26^.

We confirmed the specificity of this assay in 221 cells infected *in vitro* with SHIV_AD8OE._ As in the SIV TILDA, all p27+ cells were positive for the SHIV TILDA and none of the p27− cells. In contrast, *gag*RT-qPCR could detect also p27− cells, consistent with earlier results (Fig. S2).

After validation, we decided to use the assay in an ongoing study to start investigating its possible applications and utility.

As described more in details in^44^, 3 groups of female rhesus macaques (9 macaques/each) were treated with a combination of VRC01 and anti-α_4_β_7_ antibody (respectively 10 mg/kg and 25 mg/kg), VRC01 alone or control IgGs 3 days before initiation of weekly vaginal challenges with SHIV_AD8OE._ Challenges were performed until all animals in the 3 groups were infected in order to study the impact of the treatment/s on acute and early-chronic infection. Treatments with the anti-α_4_β_7_ antibody were repeated every 3 weeks for a total of 6 infusions. Treatment with the combination VRC01+ anti-α_4_β_7_ significantly delayed infection acquisition, preserved CD4 counts and modified other virological and immunological parameters.

**Figure 6:**
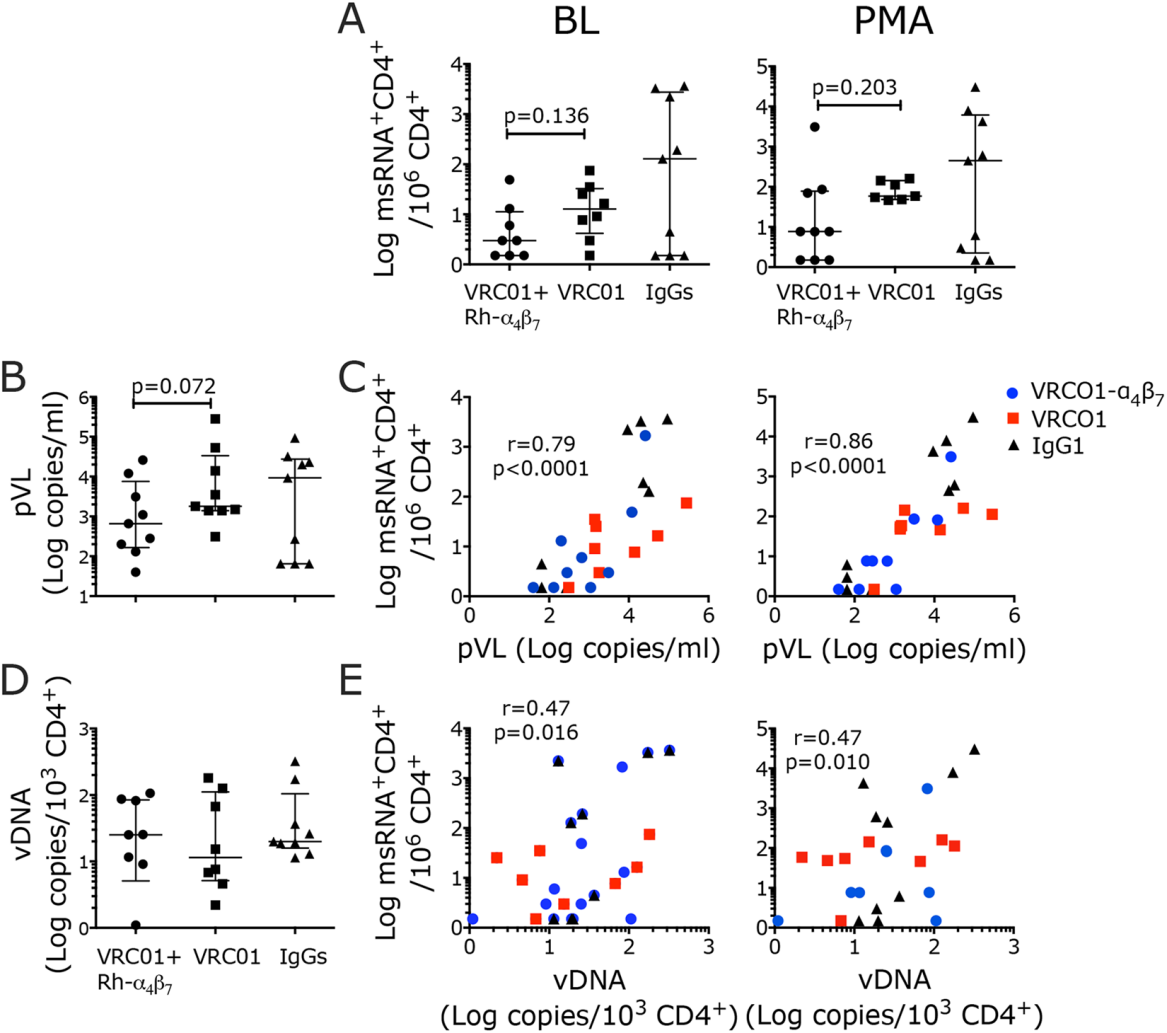
SHIV TILDA measures the frequency of CD4^+^ T cells with inducible msRNA in the lymph nodes of SHIV_AD8OE_ infected macaques. CD4^+^ T cells were isolated from the mesenteric lymph nodes of SHIV_AD8OE_ infected macaques at necropsy (~week 20 p.i.) and the SHIV TILDA assay was run on freshly isolated cells. (**A**) The frequency of CD4^+^ T cells producing msRNA in the unstimulated (BL) and stimulated (PMA) conditions are shown by treatment group (circles for VRC01-α_4_β_7_, squares for VRC01 alone and triangles for controls). (**B**) pVL at the time of necropsy is shown by treatment group. (**C**) The frequency of CD4^+^ T cells producing msRNA in the unstimulated (left) and stimulated (right) conditions are plotted against the pVL at the same time point of necropsy (**D**) Copies of CA- SHIV DNA per 10^3^ isolated lymph node CD4^+^ T cells are shown. (**E**) The frequency of CD4^+^ T cells producing msRNA in the unstimulated (left) and stimulated (right) conditions are plotted against the copies of CA- SHIV DNA per 10^3^ CD4^+^ T cells. median ± IQR. Data from the treatment groups were compared with the control by Kruskal-Wallis test and the results of the Dunn’s multiple comparisons post-hoc test and the Mann-Whitney test to compare the treatment groups between each other are shown. Correlations were calculated using the non-parametric Spearman test. All data were included. Spearman r and p values are shown (p < 0.05 was considered significant). *p*-value of *α < 0.05 and **α < 0.01 were considered significant.

Since the anti-α_4_β_7_ blocks the interaction between integrin α_4_β_7_ and its natural ligand MAdCAM-1, which is critical for trafficking of lymphocytes to the Payers patches and mesenteric lymph nodes (MLN), we explored the use of the SHIV TILDA in the MLNs of VRC01+ anti-α_4_β_7_ treated macaques to compare with VRC01-alone and control groups. Interestingly, in the control macaques the SHIV data were split in 2 groups with high and low amounts of msRNA in both the stimulated and unstimulated conditions (Fig. 6A). This mimicked the split in a high and low group according to their pVL (Fig. 6B). In contrast, the SHIV TILDA data in the treatment groups grouped together and there was a tendency toward lower frequencies of msRNA in the VRC01+ anti-α_4_β_7_ compared with the VRC01 group, which did not reach statistical significance as in the pVL (Fig. 6A,B). Of note, the frequency of msRNA in the stimulated sample would be significantly different in the 2 treatment groups in absence of the 2 outliers (as identified by the ROUT test: the highest SHIV TILDA value in the VRC01+ anti-α_4_β_7_ and the lowest in the VRC01-alone group; p = 0.018).

As expected, when the TILDA data from all 3 groups were pooled, there was a significant correlation between pVL and frequency of msRNA in both unstimulated and stimulated condition (Fig. 6C). However, no correlation was observed if the groups were considered separately (not shown).

In contrast to the pVL and SHIV-TILDA results, the amount of CA- SHIV DNA as measured by standard *gag-*qPCR on the purified CD4^+^ T cells from the MLNs was very similar in all 3 groups (Fig. 6D). Moreover, the frequency of CD4^+^ T cells expressing msRNA at baseline or after stimulation poorly correlated with the amount of CA-SHIV DNA (Fig. 6E).

## Discussion

Combination antiretroviral therapy (cART) effectively suppresses viral replication and prevents disease progression in HIV infected individuals. However, the early establishment of latent infection within resting CD4^+^ T cells and the presence of poorly characterized viral reservoirs in tissues represent major obstacles to a definitive cure for HIV. NHP models of HIV infection have been essential to elucidate pathogenic processes and constitute an important tool to test novel therapeutic strategies before they reach the clinic. Several different assays have been developed to measure residual viral replication and the extent of the viral reservoir in HIV infected patients. Their applicability to the NHP models is often constrained by their limitations (requirement for large numbers of cells and over or underestimation of the viral reservoir) and by intrinsic differences between the NHP models and cART suppressed HIV infection: in macaque models cART can be initiated very early after infection, while this rarely occurs in humans and length of cART is usually months in NHP studies, while humans are generally treated for years before other interventions are tested. These differences may lead to a smaller latent reservoir, possibly not yet in a steady state, in the context of higher residual viral replication (especially in tissues) in macaques than in humans. Indeed, although a direct comparison between data from the HIV TILDA and our data is not possible due to the differences in the type of samples used^26^, the lower fold increase in msRNA after stimulation in the macaques supports the assumption of a smaller latent reservoir.

The availability of novel assays to measure residual viral replication and reservoirs in NHP models may help better characterize viral dynamics and determine the effect of novel therapeutic strategies in NHP models. Herein, we describe a tat/rev induced limiting dilution assay to measure the frequency of CD4^+^ T cells that express msSIV/ SHIV RNA in presence and in absence of maximal stimulation. It is known that this stimulation may not lead to the reactivation of all latently infected cells harboring replication competent viruses^24^. Nonetheless, additional rounds of stimulation may increase the chances of killing infected cells with intact proviral DNA^20^ before msRNA could be detected. Thus, the SIV/SHIV TILDA assay, as the HIV TILDA assay, may, at the same, time overestimate and underestimate the size of the latent reservoir. However, considering the abovementioned differences between humans and NHP studies, a precise estimate of the latent reservoir may be less critical in NHP studies than in the clinic and should always be considered in the context of other measures of viral burden that encompass the whole body including residual viremia in tissues^14^ and data from novel whole-body imaging techniques such as positron-emission tomography (PET)-based imaging of the SIV reservoir^16^.

The SIV/SHIV assay was validated using cell lines infected with SIVmac239 and SHIV_AD8OE_ and in cells from blood and lymph nodes of macaques infected with SIVmac239, SIVmac251 and SHIV_AD8OE_ in presence and absence of cART. *In vitro* and *ex vivo*, SIV/SHIV TILDA detects only cells also expressing viral proteins confirming that the presence of tat/rev transcripts reflects the ability of a cell to produce virus. Indeed, although TILDA does not bypass completely the possibility of measuring defective proviruses^29^, a large fraction of defective proviruses appears to have deletions encompassing the tat/rev region^24^. Of note, the RT reaction in the SIV TILDA, as in the HIV TILDA is done directly without RNA extraction. This may be possible because of increased permeability/lysis of the lymphocytes at the 50 °C needed for the RT reaction to occur. However, this hypothesis needs further investigation.

In SIV/SHIV infected macaques, CD4^+^ T cells that express msSIV/SHIV RNA were detected also in the setting of very low viremia, both in presence an absence of stimulation, but only in 1 out of 4 LNs from macaques with undetectable viremia that had been treated for months with suppressive cART. This may be due to an issue of assay sensitivity or simply be the result of months of treatment in these macaques. More studies involving cART-treated macaques with undetectable viremia are needed to distinguish between these possibilities. Importantly, the development of new therapeutic strategies for NHP models of HIV infection^45^ made long, suppressive treatments in NHP feasible, although not common because of the high costs. Thus, the performance of the SIV TILDA assay will have to be evaluated in these models of long-term fully suppressed viremia, who truly mimic HIV infected patients treated with cART for years. The limitation of the assay in these models will probably be the sensitivity of infected cells to PMA/Ionomycin activation. The frequency of cell death resulting from the activation step may be higher than the frequency of CD4^+^ T cells expressing msRNA detectable by TILDA.

As expected, TILDA frequencies were significantly higher after stimulation than in absence of stimulation and TILDA data correlated with pVL. However, correlation with pVL was significant only if a large number of samples were included, which suggests an overall poor association between the 2 measurements. Importantly, no significant correlation was found between CA- SHIV DNA in LNs CD4^+^ T cells and either unstimulated or stimulated frequency of CD4^+^ T cells expressing msRNA. This association was not tested in blood CD4^+^ T and the CA-DNA in PBMC was not measured. Considering that CA-DNA in PBMC at the time of treatment interruption was found to predict post-cART viral control^46^, it will be important to determine if SIV/SHIV TILDA correlates with CA-DNA in PBMC as it is the case for HIV TILDA^26^. Importantly, the lack of correlation of the CA-DNA with the SIV TILDA data found in the LNs may be due to the instability of the reservoir in viremic RMs and differences between viremic and fully suppressed macaques should be explored in future work. Nonetheless, we demonstrated that the SIV/SHIV TILDA may be able to detect differences in viral burden (productive and latent) between treatment groups, such as those between LNs from VRC01 and VRC01-α_4_β_7_ treated macaques, that were not apparent when comparing CA-SHIV DNA.

Interestingly, TILDA data from early after cART initiation significantly correlated with two different measures of post-treatment virologic control, peak and AUC plasma viral load of the first 5 weeks after treatment interruption. Although time to rebound is a more classically used metrix to evaluate the effect of interventions that aim to decrease the viral reservoir in cART treated individuals^19^, in cases like ours where time-to-rebound does not differ substantially between macaques, early post-interruption pVL may be a good indicator of the size of the viral reservoir during therapy. Of note, pVL at the time of TILDA did not correlate with post-treatment virologic control. Therefore, the TILDA assay constitutes an important additional source of information on residual viremia and viral reservoir than only pVL. However, issues of instability of the viral reservoir early after cART initiation and the small sample size of our study do not allow for an accurate interpretation of this correlation and future studies with a larger number of completely suppressed animals should be performed to explore the ability of the SIV TILDA to predict viral rebound. The size of the expressed HIV reservoir was found to predict viral rebound after treatment interruption^47^. In SIV-infected macaques early after cART initiation, when virologic suppression is not yet achieved, TILDA data may reflect the “size of expressed reservoir” better than other measurements such as CA-viral DNA (vDNA) or CA-viral RNA (vRNA). However, CA-vDNA/RNA were not measured in the cART treated macaques in our study. Thus, a direct comparison between the assays cannot be made.

In conclusion, the SIV/SHIV TILDA assay could be useful in measuring the viral reservoir in medium/low-viremic animals that control spontaneously or soon after cART initiation. Its utility and sensitivity in the context of undetectable viremia needs further evaluation. This assay in combination with other molecular, culture-based and imaging techniques will be useful to investigate the viral reservoirs in NHP models. It may be particularly suitable to characterize tissue reservoirs and help assessing the effect of novel intervention strategies that aim either at viral eradication or at a functional cure for HIV.

## Materials and Methods

### Isolation of CD4^+^ T cells

CD4^+^ T cells were isolated from blood or lymph nodes (LNs). PBMCs were isolated from blood using Ficoll-Hypaque density gradient centrifugation. LNs were cut in small pieces, passed through a 70 μm cell strainer and rinsed with cold PBS. CD4^+^ T cells were isolated (from fresh PBMC and mesenteric LNs (MLNs) for SHIV_AD8OE_, SIVΔNef and SIVmac251 infected animals or frozen PBMC/LNMC for SIVmac239 and SIVmac239Nef_stop infected animals) by negative selection using the non-human primate CD4^+^ T cell isolation kit (Miltenyi). Freshly enriched CD4^+^ T cells were resuspended at 2 × 10^6^ cells/ml in complete cRPMI (complete RPMI, 10% fetal bovine serum, 100 U/ml Penicillium and 100 μg/ml Streptomycin, 10 mM Hepes, 2 mM L-glutamine). A small fraction of cells was used to assess the purity of enriched CD4^+^ T cells (generally greater than 96%) and the level of T cell activation by flow cytometry.

### SIV and SHIV TILDA

Enriched CD4^+^ T cells were split in half. One half of cells was stimulated with 100 ng/ml PMA and 1 μg/ml ionomycin (both from Sigma), while the second half was kept in medium only. After 16 hours, cells were washed in cRPMI, counted and a small fraction of stimulated and unstimulated cells was used to confirm the activation or resting state of T cells, respectively. Remaining cells were then serially diluted to 18 × 10^6^ cells/ml, 9 × 10^6^ cells/ml, 3 × 10^6^ cells/ml and 1 × 10^6^ cells/ml (or otherwise as indicated) in cRPMI. 1 μl/well of each dilution of stimulated or unstimulated CD4^+^ T cells was then distributed in 22 wells of a 96 well PCR plate already containing 10 μl/well of PCR reaction mix (1 PCR plate for stimulated and 1 PCR plate for unstimulated T cells) corresponding to 18,000, 9000, 3000 or 1000 cells/well. As indicated in the results, the cell number used in macaques with higher pVL was 9000, 3000, 1000, 300 or 3000, 1000, 300, 100. The PCR reaction mix for one reaction consisted of 5 μl of 2X reaction buffer of the SuperScript III Platinum One-Step qRT-PCR Kit (Life Technologies), 0.2 μl Superscript III Platinum Taq (Life Technologies), 0.3 μl RNase inhibitor (Promega), 0.125 μl of each primer (for SIVmac239: SIV*tat/rev*FW and SIV*tat/rev*REV; for SHIV_AD8OE_: SIV*tat/rev*REV and HIV*tat/rev*1-2 REV; both at 20 μM), 2.2 μl Tris–EDTA (TE) buffer and 2.05 μl nuclease-free water (final reaction volume 11 ul/well). The sequences of the primers used for SIVmac239 were: SIV*tat/rev*FW 5′-CAC GAA AGA GAA GAA GAA CTC CG-3′ and SIV*tat/rev*REV 5′-TCT TTG CCT TCT CTG GTT GG-3′, and for SHIVAD8 the SIV*tat/rev*FW 5′-CAC GAA AGA GAA GAA GAA CTC CG-3′ and the HIV*tat/rev*1-2 REV 5′-GGA TCT GTC TCT GTC TCT CTC TCC ACC-3′ which is identical to the HIV TILDA assay, adapted from^33^. Pre-amplification using whole cells included reverse transcription at 50 °C for 15 min, denaturation at 95 °C for 2 min, and 24 cycles of amplification at 95 °C for 15 sec and 60 °C for 4 minutes in a Biorad Thermal Cycler (MyCycler or ICycler). Following pre-amplification 1 μl of each PCR product was used as template for the ***tat***/***rev*** real-time PCR reaction and distributed to a 96 well PCR already containing 9 μl of Taqman qPCR reaction mix consisting of 5 μl 2X reaction buffer of the Taqman Universal master mix II (Thermo Fisher), 0.2 μl of each primer (for SIVmac239: SIV *tat/rev* Nested FW Set1 and SIV*tat/rev*REV; for SHIVAD8: SIV tat/rev Nested FW Set1 and HIV tat/rev1-2 REV; both at 20 uM), 0.2 μl of SIV or HIV FamZen probe at 5 μM, and 3.4 μl nuclease-free water to each well (final reaction volume 10 μl/well). The SIV FamZen probe (SIV tat/rev P) was: 5′-/56-FAM/AA ACC CAT A/ZEN/T CCA ACA GGA CCC GG/3IABkFQ/-3′, and the = HIV FamZen probe (HIV*tat*/*rev*P at 5 uM) from^33^, was: 5′-/56-FAM/TTC CTT CGG/ZEN/GCC TGT CGG GTC CC/3IABkFQ/-3′. All primers and probes were synthesized by IDT. The real-time PCR reaction was carried out with a ViiA7 Real-Time PCR Instrument (Thermo Fisher) using the following program: preincubation 95 °C for 10 min, 45 cycles of 95 °C 15 s, 60 °C 1 min, and a cooling step at 40 °C. Positive wells at each dilution were counted and the maximum likelihood method was used to calculate the frequency of CD4^+^T cells expressing msRNA (http://bioinf.wehi.edu.au/software/elda).

### SIV TILDA Specificity and Sensitivity

To determine the specificity of the SIV TILDA CEMX174 cells were infected with SIVmac239 or 221 cells (kindly gifted by Prof Desrosiers^48^) were infected with SHIV_AD8OE_ for 3 hours at 37 °C before virus was washed out and cells were resuspended in cRPMI supplemented with 50 U/ml of IL-2. Three days post infection CEMX174 or 221 cells were stained with the LIVE/DEAD Aqua dye (Invitrogen), fixed and permeabilized with the Fix/Perm kit (BDBioscience) for 30 minutes at 4 °C and then stained with a DyeLight 488 conjugated anti-SIV Gag p27 antibody (clone 55-2F12 from the NIH AIDS reagents repository and conjugated with the Innova Bioscience kit) diluted in Perm/Wash buffer (BDBioscience) for 20 minutes at room temperature. The p27 antibody was tittered in a separate experiment on SIVmac239 infected CEMX174 cells and SHIV_AD8OE_ infected 221 cells. Excess antibody was removed by washing cells three times with cold Perm/Wash buffer and PBS. Thereafter, cells were resuspended in cold PBS and passed through a test tube with cell strainer snap cap (Fisher Scientific) to clear samples of possible cell aggregates before p27 positive or p27 negative cells were sorted on a BD FACS Aria II cell sorter (Rockefeller Flow Cytometry Resource Center) 1–10 cells/well in 47 or 50 replicates per plate in a 96-well PCR plate already containing PCR reaction mix. To ensure the collection of 100% p27 negative or 100% p27 positive (high) cells, sort gates were set very conservative. Two plates per cell line, 1 plate with p27 negative CEMX174 or 221 cells and 1 plate with p27 positive CEMX174 or 221 cells, were used for pre-amplification of *tat/rev* msRNA followed by real-time PCR using SIV or SHIV specific primers and probes exactly as described above for the TILDA assay. Two additional plates with p27 negative or p27 positive CEMX174 or 221 cells were used to perform a SIV*gag* One-Step RT-PCR (SuperScript III Platinum). For this, PCR plates were preloaded with 19 μl/well of PCR reaction mix that consisted of 10 μl 2X reaction buffer of the SuperScript III Platinum One-Step qRT-PCR Kit, 0.4 μl Superscript III Platinum Taq, 0.6 μl RNase inhibitor, 0.25 μl of each primer (for SIVmac239 and SHIVAD8: SIVgagF and SIVgagR; both at 20 μM), 0.25 μl of SIV FamZen probe (SIVgagP at 10 uM), 4.4 μl Tris–EDTA (TE) buffer and 2.85 μl nuclease-free water. The sequences for SIVgag primers and probe were previously published^49^. Real-time PCR was performed on a ViiA7 Real-Time PCR Instrument with the following program: reverse transcription 50 °C for 15 min, enzyme activation 95 °C for 15 sec, 40 cycles of 95 °C for 15 sec, 60 °C for 4 min, and a cooling step at 4 °C. To determine the specificity of the SIV TILDA in CD4^+^ T cells from PBMC of SIV chronically infected macaques, frozen PBMC were thawed and CD4^+^ T cells isolation performed after 2 hrs of resting in R10. CD4^+^ T cells were stimulated with 100 ng/ml PMA and 1 μg/ml ionomycin as in the SIV TILDA stimulation in presence of T20 (200 nM). After O/N incubation, PMA/ ionomycin were removed by washing and cells cultured for additional 2 days to allow for production of viral particles. P27 ICS and sorting were performed as described above for the CEMX174 and 221 cells.

Evaluation for the sensitivity of the semi-nested RT-qPCR was performed on SIV infected CD4^+^ macaque T cells. For this, CD4^+^T cells were activated with anti-CD3/CD28 antibodies before being infected with SIVmac239. 100% infection was confirmed by staining cells for the SIV p27 protein as described above. SIV-infected cells were then serially diluted in 10-fold steps ranging from 1 × 10^6^ cells/ml down to 1 × 10^3^ cells/ml and mixed with uninfected CD4 T cells at 10^7^ cells/ml. Thereafter, 1 μl/well of each mix of infected and uninfected cells (which only varied in the number of infected cells) was distributed in a 96-well PCR plate that already contained 10 μl/well of PCR reaction mix. For each condition a total of 10 replicates was setup. Final numbers of infected CD4^+^ T cells per well were 1000, 100, 10 and 1, each in a background of 10,000 uninfected CD4 T cells. Pre-amplification and real-time PCR to detect ***tat***/***rev*** msRNA was carried out as described above.

### SIV and SHIV infected animals

PBMC and LNMC samples were collected from the Indian rhesus macaques listed in Table 1 and S1. All animals were Mamu-A*01, Mamu-B*08 and Mamu-B*17 negative with the exception of A*01+: RIp10, RZp11, RKt9 and the SIVΔNef with unknown MHC typing. All macaques were enrolled in various studies and infected with the virus and for a time indicated in the table. Studies including the animals infected with SIVmac239 wild-type were published in^50^, while those infected with SIVmac239Nef_stop were unpublished. Animals in these studies were housed and maintained at the Yerkes National Primate Research Center (YNPRC) in accordance with the rules and regulations of the Committee on the Care and Use of Laboratory Animal Resources. All the procedures were reviewed and approved by the Emory University IACUC. The study including the SHIV_AD8OE_ infected animals was published in (Calenda *et al*. submitted). SHIV_AD8OE_, SIVΔNef and SIVmac251 infected animals were housed, indoors, in climate controlled conditions at the Tulane National Primate Research Center (TNPRC) in compliance with the regulations under the Animal Welfare Act, the Guide for the Care and Use of Laboratory Animals^51^. All procedures in these animals were approved by the Animal Care and Use Committee of the TNPRC and in compliance with the Animal Welfare Act and the Guide for the Care and Use of Laboratory Animals.

### Statistics

Data in the BL and PMA conditions were compared with the Wilcoxon matched pairs signed-rank test (α = p < 0.05 was considered significant). Correlations were assessed using the non-parametric Spearman test. Outliers were not excluded. In the studies with SHIV_AD8OE_ infected macaques, the VRC01 and VRC01-α_4_β_7_ treatment groups were compared directly with a two-tailed unpaired nonparametric Mann-Whitney test (α = p < 0.05 was considered significant). Each treatment group was compared to the control group by Kruskal-Wallis test with Dunn’s multiple comparison test as post-hoc test. Analysis was performed with the Prims 7 software (GraphPad). To test for outliers in the correlations and comparisons, we used an adaptation of the ROUT method^52^ in Prism 7 that allows the use of this test with data in columns/groups. Data were interpreted with caution because the ROUT test assumes Gaussian distribution. This assumption is not valid with small data sets as ours.

## Supplementary information

Supplementary Information

## Data Availability

All data associated with the current study are available from the corresponding author upon request.
