# Supplementary material for: A Tat/Rev Induced Limiting Dilution Assay to Measure Viral Reservoirs in Non-Human Primate Models of HIV Infection
Source: Sci Rep. 2019 Aug 19;9:12078. doi: 10.1038/s41598-019-48354-3 (PMC6700126; doi:10.1038/s41598-019-48354-3)
Supplement: Supplementary file 1 — Supplementary Information [file 41598_2019_48354_MOESM1_ESM.pdf]

Supplementary Information:

A Tat/Rev Induced Limiting Dilution Assay to Measure Viral Reservoirs in Non-Human Primate Models of HIV Infection.

Ines Frank[1], Arpan Acharya[2], Nanda K. Routhu[2], Meropi Aravantinou[1], Justin L. Harper[3] Stephanie Maldonado[1], Maria Sole Cigoli[1], Stanka Semova[4], Svetlana Mazel[4], Mirko Paiardini[3], Nina Derby[1], Siddappa N Byrareddy[2], and Elena Martinelli[1∞].

[1]Center for Biomedical Research, Population Council, New York, NY, USA; [2]Department of Pharmacology and Experimental Neurosciences, University of Nebraska Medical Center, Omaha, USA; [3]Yerkes National Primate Research Center, Emory University, Atlanta, Georgia, USA; [4]Flow Cytometry Resource Center, Rockefeller University, New York, NY, USA.

Fig S1

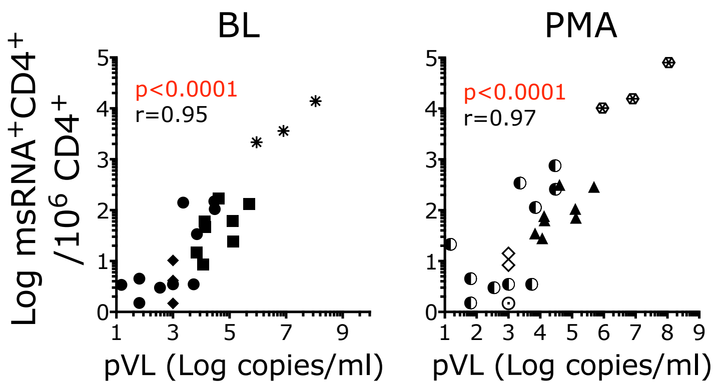

Figure S1. The frequency of $CD4^+$ T cells producing msRNA in SIV infected macaques correlates with pVL. Frequency of $CD4^+$ T cells expressing msRNA per 10^6 $CD4^+$ T cells in the unstimulated (BL) and stimulated (PMA) conditions is plotted against the plasma viral load at the time of the assay. Data from all SIV infected macaques are shown (including macaques with high and low/undetectable viremia). Correlation was calculated using the non-parametric Spearman test. No outlier was excluded. Spearman r and p values are shown ($p<0.05$ was considered significant).

Fig S2

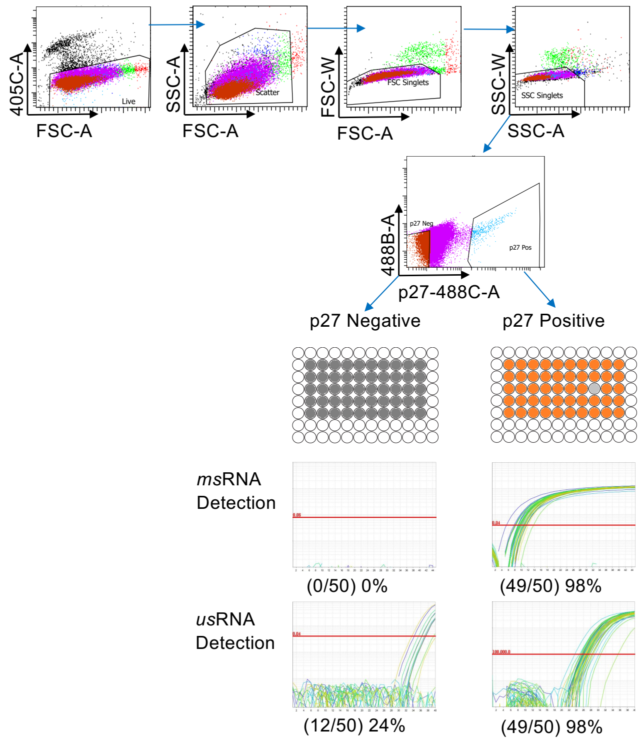

Figure S2. The SHIV TILDA detects msRNA only in cells expressing also viral proteins. 221 cells were infected with $SHIV_{AD8OE}$ for 3 days and then p27+ and p27- cells were sorted on a BD FACS Aria II cell sorter directly into 50 wells (10 cells/each) of a 96 well plate containing mastermix. TILDA semi-nested RTqPCR or *gag*RT-qPCR were run. Sorting gating strategy is shown above, while actual amplification curves from the assays are shown below next to the final results.

Table S1. List of macaques used in the study with the SHIV TILDA. Detailed characteristics of each animals are listed.

Macaque	Sex	Virus	Week p.i.	Plasma VL	Sample Type	cART Regiment	Treatment
EM09	F	$SHIV_{AD8OE}$	19	12000	MLN	n/a	VRC01 + Rh-α4β7
GH63	F	$SHIV_{AD8OE}$	18	1100	MLN	n/a	VRC01 + Rh-α4β7
HB73	F	$SHIV_{AD8OE}$	21	40	MLN	n/a	VRC01 + Rh-α4β7
HN68	F	$SHIV_{AD8OE}$	21	130	MLN		VRC01 + Rh-α4β7
IB76	F	$SHIV_{AD8OE}$	21	660	MLN	n/a	VRC01 + Rh-α4β7
JD62	F	$SHIV_{AD8OE}$	20	200	MLN	n/a	VRC01 + Rh-α4β7
KT57	F	$SHIV_{AD8OE}$	18	280	MLN	n/a	VRC01 + Rh-α4β7
FH61	F	$SHIV_{AD8OE}$	18	26000	MLN	n/a	VRC01 + Rh-α4β7
JJ39	F	$SHIV_{AD8OE}$	23	3100	MLN	n/a	VRC01 + Rh-α4β7
CJ36	F	$SHIV_{AD8OE}$	22	53000	MLN	n/a	VRC01
CT02	F	$SHIV_{AD8OE}$	20	3500	MLN	n/a	VRC01
ED86	F	$SHIV_{AD8OE}$	18	14000	MLN	n/a	VRC01
FT58	F	$SHIV_{AD8OE}$	23	1800	MLN	n/a	VRC01
HF66	F	$SHIV_{AD8OE}$	21	310	MLN	n/a	VRC01
IK06	F	$SHIV_{AD8OE}$	22	1500	MLN	n/a	VRC01
KT03	F	$SHIV_{AD8OE}$	20	1400	MLN	n/a	VRC01
EN78	F	$SHIV_{AD8OE}$	20	280000	MLN	n/a	VRC01
HI27	F	$SHIV_{AD8OE}$	23	1400	MLN	n/a	VRC01
HR40	F	$SHIV_{AD8OE}$	19	270	MLN	n/a	IgGs
IM69	F	$SHIV_{AD8OE}$	19	65	MLN	n/a	IgGs
IV98	F	$SHIV_{AD8OE}$	20	65	MLN	n/a	IgGs
JV32	F	$SHIV_{AD8OE}$	19	32000	MLN	n/a	IgGs
KP80	F	$SHIV_{AD8OE}$	20	9300	MLN	n/a	IgGs
HM14	F	$SHIV_{AD8OE}$	21	20000	MLN	n/a	IgGs
HN46	F	$SHIV_{AD8OE}$	21	65	MLN	n/a	IgGs
IJ84	F	$SHIV_{AD8OE}$	20	23000	MLN	n/a	IgGs
IK53	F	$SHIV_{AD8OE}$	20	93000	MLN	n/a	IgGs
